# Supplementary material for: Conserved heavy/light contacts and germline preferences revealed by a large-scale analysis of natively paired human antibody sequences and structural data
Source: Commun Biol. 2025 Jul 26;8:1110. doi: 10.1038/s42003-025-08388-y (PMC12297541; doi:10.1038/s42003-025-08388-y)
Supplement: Supplementary file 1 — Supplementary Information [file 42003_2025_8388_MOESM1_ESM.pdf]

Supplementary table 1. Number of heavy/light chain sequences and Light chain proportions for each organism.

organism	igh	igk	igl	igk_ratio	igl_ratio
human	6566748	3959197	2607551	0.603	0.397
mouse	633886	570298	63588	0.9	0.1

Supplementary table 2. Number of heavy /light chain sequences and Light chain proportions for each human project.

bioproject	organism	igh	igk	igl	igk_ratio	igl_ratio
PRJEB50691	human	57005	35438	21567	0.62	0.38
PRJNA1011870	human	1052	579	473	0.55	0.45
PRJNA998068	human	2481	1818	663	0.73	0.27
PRJNA727275	human	23444	14716	8728	0.63	0.37
PRJNA1014484	human	3444	2379	1065	0.69	0.31
PRJNA847159	human	47238	30340	16898	0.64	0.36
PRJNA782883	human	80491	46714	33777	0.58	0.42
PRJNA997992	human	45076	30561	14515	0.68	0.32
PRJNA913893	human	19539	12379	7160	0.63	0.37
PRJNA670581	human	123988	75118	48870	0.61	0.39
PRJNA1014462	human	13040	7538	5502	0.58	0.42
PRJEB51634	human	203455	137654	65801	0.68	0.32
PRJNA855895	human	501679	303292	198387	0.60	0.40
PRJNA742201	human	21628	15045	6583	0.70	0.30
PRJEB52699	human	25350	12864	12486	0.51	0.49
PRJEB47921	human	14641	8996	5645	0.61	0.39
PRJNA883612	human	70017	47927	22090	0.68	0.32
PRJNA927026	human	45322	25373	19949	0.56	0.44
PRJNA978638	human	4172	2733	1439	0.66	0.34
PRJNA1024473	human	10079	6806	3273	0.68	0.32
PRJNA907025	human	1041109	643959	397150	0.62	0.38
PRJNA1073945	human	12806	7754	5052	0.61	0.39
PRJNA610059	human	78419	46290	32129	0.59	0.41
PRJEB53053	human	582884	294884	288000	0.51	0.49
PRJNA717310	human	21623	11631	9992	0.54	0.46
PRJEB61178	human	48806	33896	14910	0.69	0.31
PRJEB63391	human	25024	15470	9554	0.62	0.38

PRJNA744567	human	13754	6949	6805	0.51	0.49
PRJNA803769	human	6351	3751	2600	0.59	0.41
PRJNA777934	human	844204	498227	345977	0.59	0.41
PRJNA962178	human	296086	170211	125875	0.57	0.43
PRJNA988459	human	19252	9039	10213	0.47	0.53
PRJEB52996	human	3561	1993	1568	0.56	0.44
PRJNA953326	human	2457	1778	679	0.72	0.28
PRJEB54608	human	31873	20075	11798	0.63	0.37
PRJNA975614	human	5866	3245	2621	0.55	0.45
PRJNA898288	human	114096	77046	37050	0.68	0.32
PRJNA908079	human	56042	30959	25083	0.55	0.45
PRJNA800176	human	670874	450327	220547	0.67	0.33
PRJEB40825	human	349580	203493	146087	0.58	0.42
PRJNA958150	human	234	124	110	0.53	0.47
PRJNA961775	human	32452	18556	13896	0.57	0.43
PRJNA975506	human	485941	307564	178377	0.63	0.37
PRJNA512111	human	510313	283706	226607	0.56	0.44

Supplementary table 3. Grouped metrics of chain proportions of sequences from human projects.

metric	igh	igk	igl	igk_ratio	igl_ratio
mean	149244,27	89981,75	59262,52	0,61	0,39
std	249344,59	150902,55	99799,53	0,06	0,06
min	234,00	124,00	110,00	0,06	0,27
max	1041109,00	643959,00	397150,00	0,73	0,53

Supplementary table 4. Chain proportions for each mouse project.

bioproject	organism	igh	igk	igl	igk_ratio	igl_ratio
PRJNA980254	mouse	2109	1942	167	0.92	0.08
PRJNA921879	mouse	3649	3466	183	0.95	0.05
PRJNA844903	mouse	1694	1653	41	0.98	0.02
PRJEB49019	mouse	173847	161748	12099	0.93	0.07
PRJNA845790	mouse	4735	4496	239	0.95	0.05
PRJEB34451	mouse	6883	6679	204	0.97	0.03
PRJNA777966	mouse	70770	66843	3927	0.94	0.06
PRJNA990769	mouse	3161	2700	461	0.85	0.15
PRJNA1082281	mouse	81818	54478	27340	0.67	0.33

PRJNA934380	mouse	15284	14710	574	0.96	0.04
PRJEB64695	mouse	144667	131659	13008	0.91	0.09
PRJNA1026914	mouse	1427	1388	39	0.97	0.03
PRJEB50082	mouse	12044	11182	862	0.93	0.07
PRJNA839019	mouse	111798	107354	4444	0.96	0.04

Supplementary table 5. Grouped metrics of chain proportions of sequences from mouse projects.

metric	igh	igk	igl	igk_ratio	igl_ratio
mean	45277,00	40735,00	4542,00	0,92	0,08
std	60213,00	55233,00	7897,77	0,08	0,08
min	1427,00	1388,00	39,00	0,67	0,02
max	173847,00	161748,00	27340,00	0,98	0,33

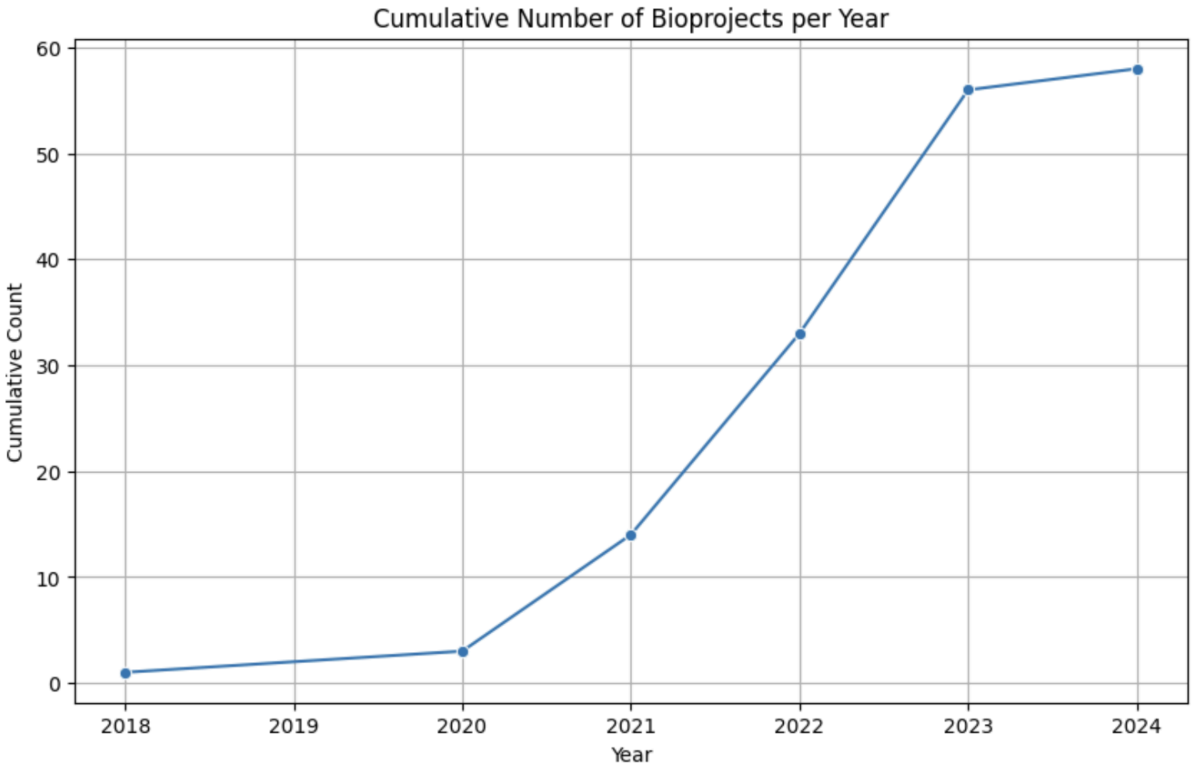

Supplementary figure 1. Cumulative number of single-cell (paired heavy/light) bioprojects published per year. Please note since the identification was performed

in May 2024, the numbers for 2024 are incomplete and are therefore the increment is lower than for previous years.

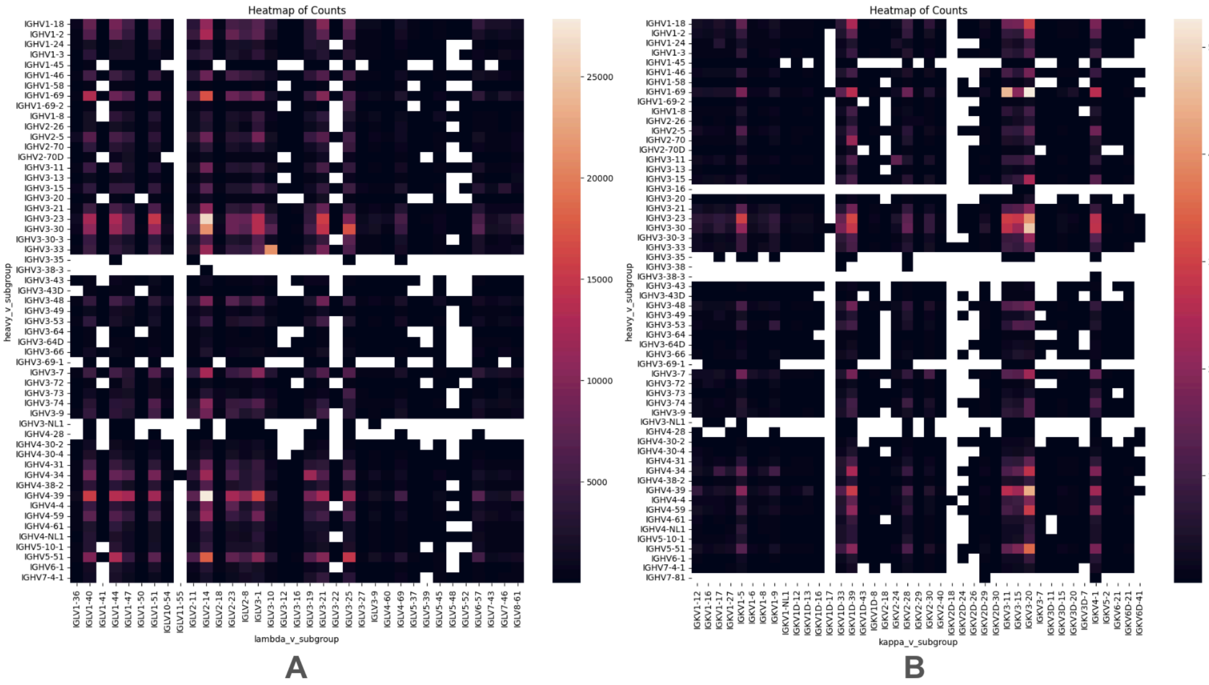

Supplementary figure 2. V gene subgroup pairing counts. A. Lambda chains. **B.** Kappa chains. Heatmaps represent observed counts of V gene subgroup pairs in heavy and light chains.
