# Supplementary material for: Conserved heavy/light contacts and germline preferences revealed by a large-scale analysis of natively paired human antibody sequences and structural data
Source: Commun Biol. 2025 Jul 26;8:1110. doi: 10.1038/s42003-025-08388-y (PMC12297541; doi:10.1038/s42003-025-08388-y)
Supplement: Supplementary file 2 — Description of Additional Supplementary files [file 42003_2025_8388_MOESM2_ESM.pdf]

Description of Additional Supplementary files

File name: Supplementary Data 1

Supplementary Data 1 contains the numerical data for all figures
